# Lithium Phosphorus Sulfide Chloride–Polymer Composite via the Solution–Precipitation Process for Improving Stability toward Dendrite Formation of Li-Ion Solid Electrolyte

**DOI:** 10.1021/acsami.2c21302

**Published:** 2023-02-24

**Authors:** Piyachai Khomein, Young-Woon Byeon, Dongye Liu, Jin Yu, Andrew M. Minor, Haegyeom Kim, Gao Liu

**Affiliations:** † Division of Nuclear Medicine, Department of Radiology, Faculty of Medicine, Chulalongkorn University, Bangkok 10330, Thailand; ‡ Energy Storage and Distributed Resources Division, Energy Technologies Area, Lawrence Berkeley National Laboratory, Berkeley, California 94720, United States; § Materials Sciences Division, Lawrence Berkeley National Laboratory, Berkeley, California 94720, United States; ∥ Department of Materials Science and Engineering, University of California, Berkeley, California 94720, United States; ⊥ Department of Chemical & Biomolecular Engineering, University of California, Berkeley, California 94720, United States; # National Center for Electron Microscopy, The Molecular Foundry, Lawrence Berkeley National Laboratory, Berkeley, California 94720, United States

**Keywords:** polymer composite, lithium phosphorus sulfide chloride, solid-state electrolyte, dendrite blocking, low-temperature process

## Abstract

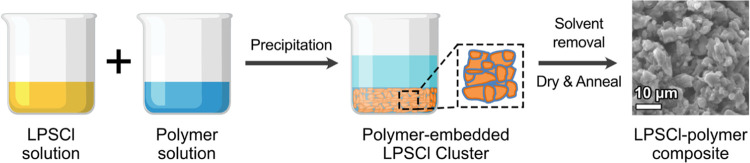

Improving the mechanical strength of ceramic solid electrolytes such as lithium phosphorus sulfide families for pressure-driven dendrite blocking as well as reducing the electronic conductivity to prevent a dendrite formation inside the electrolytes are very important to extend the lifespan of all-solid-state lithium-metal batteries. Here, we propose a low-temperature solution–precipitation process to prepare polymer–solid electrolyte composites for a highly uniform polymer distribution in the electrolyte to enhance their mechanical strength and reduce their electronic conduction. The composites with up to 12 wt % of polymer are prepared, and the composites exhibit high ionic conductivities of up to 0.3 mS/cm. Furthermore, the electrochemical stability of the electrolyte composites on Li striping/plating cycles is investigated. We confirm that the proposed solution–precipitation process makes the composite much more stable than the bare solid electrolyte and causes them to outperform similar composites from the other existing preparation methods, such as mechanical mixing and solution dispersion.

## 1 Introduction

All-solid-state batteries (SSBs) have been considered the next-generation energy storage system due to many advantages over organic liquid electrolyte-based batteries. For example, they inherit low fire hazards due to lacking flammable organic components.^1^ They are also promising for lower-cost manufacturing per power output ($/kWh) compared to current liquid electrolyte-based Li-ion batteries.^2^ In addition, energy density can further be improved using a Li-metal anode, which had not been successful in liquid electrolyte systems due to insuppressible Li dendrite formation. Therefore, utilizing a solid electrolyte (SE) with a Li-metal anode has been regarded as a promising way to reduce Li dendrite growth due to their high mechanical strength and high Li^+^ transference number.^3,4^

Over the past several years, there have been efforts focused on developing SE with high ionic conductivity, and the research field has experienced tremendous growth.^5^ The combination of theory and experimental works has led to the discovery of new materials such as lithium phosphorus oxynitride (Li_3_PO_4_, LiPON),^6^ Lithium Super-Ionic CONductor families (LISICONs),^7^ lithium lanthanum zirconium oxide (Li_7_La_3_Zr_2_O_12_, LLZO),^8^ and lithium phosphorus sulfide (Li*_x_*P*_y_*S*_z_*, LPS) families.^9^ However, recent reports have indicated that lithium dendrites can still form in SE and are, in fact, more easily formed in LLZO and LPS than in the liquid electrolyte.^10^ The dendrite tends to form along grain boundaries and voids in SE,^11^ and the increasing density of SE to minimize the grain boundary did not successfully prevent such a dendrite formation.^12,13^ Even in the single-crystalline LLZO system, the dendrite growth was still observed.^14^ Recently, H. Wang and C. Wang^10^ found the direct nucleation and dendrite formation inside LLZO and LPS, suggesting that the electronic conduction across the SE materials could cause the dendrite growth inside these SEs. Therefore, lowering the overall electronic conductivity of SE while maintaining its high ionic conductivity is a critical challenge for the success of all-solid-state Li-metal batteries.

In this regard, composite SE with a polymer can be a promising approach, as it was shown to improve not only its stability toward dendrite formation but also enhance its electrochemical performance including high-voltage stability,^15^ improve ion transference number,^16^ minimize interface reaction, and better mechanical properties for ease of processability and thinner separator layer.^17,18^ Typically, the composites can be prepared by mechanical mixing or dispersion of SE in the polymer solution, followed by solvent removal. For instance, a ball-milling technique was employed to prepare an LPS polymer composite (25 wt % polymers).^19^ The electrolyte with a thickness of <70 μm was successfully fabricated with ionic conductivity ranging between 0.05 and 0.1 mS/cm. However, the nonuniform distribution of polymer in SE composite is the mechanical mixing method’s primary concern, which can impact the Li-ion transport and overall mechanical properties of composites. The solution dispersion can provide better uniform distribution between polymer and SE. The 5 wt % polyethylene oxide in lithium phosphorus sulfide chloride (PEO/LPSCl) composite has been reported to improve the full cell cycling performance using LiNi_0·8_Co_0·1_Mn_0·1_O_2_ and Li metal as electrodes with a 91% capacity retention over 200 cycles.^20^ Another impressive composite consisting of poly(vinylidene fluoride) (PVDF) and LPSCl was also reported with a conductivity of 1 mS/cm for a 10 wt % PVDF composite and the thickness of 100–120 μm was successfully fabricated.^21^

**Figure 1:**
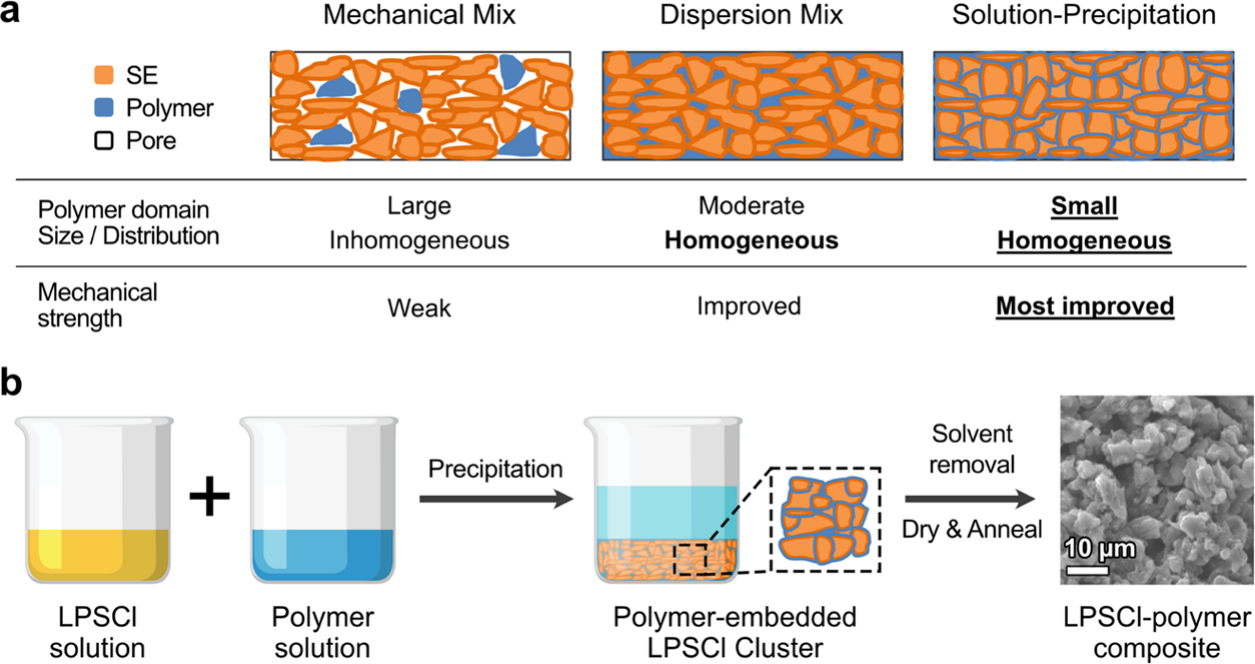
(a) Comparison of three possible methods for preparing the solid electrolyte–polymer composites. Note that the solution–precipitation method has the advantage of high mechanical strength owing to its smaller polymer domain size and uniformly distributed microstructure. (b) Schematic showing the preparation process of the solution–precipitation method by simple co-precipitation of LPSCl and polymer solutions to yield a polymer-embedded LPSCl cluster.

For the existing preparation methods for composite SE, either mechanical mixing or dispersion technique, we found that the uniformity of polymer distribution is impeded when applying a higher polymer load (>8 wt %). The phase separation could later weaken the composite’s mechanical strength, which is the critical point for pressure-driven dendrite blocking.^22^ This work proposes another approach for a new class of sulfide–polymer composite synthesis via low-temperature solution–precipitation processes (Figure 1). This method will utilize the soluble LPSCl in a polar solvent to provide a single-phase mixing for a uniformly distributed polymer composite SE to efficiently suppress the dendrite formation. Furthermore, the evenly distributed polymer can also prevent the formation of an electronic conducting interphase between SE and electrodes. Herein, we reported the synthesis of LPSCl–polymer composite (PEO and PPO) via the solution–precipitation method, which can improve the conductivity up to 0.3 mS/cm (12 wt % of polymer).

## 2 Experimental Section

### 2.1 Polymer Composites via the Solution–Precipitation Method

All chemicals were purchased from commercial sources and used as received unless mentioned otherwise. Indium foil (thickness 0.1 mm, ≥99.995% trace metals basis) was purchased from Sigma Aldrich and used as received. Battery-grade lithium foil (thickness 0.1 mm) was purchased from MSE Supplies. The lithium foil was polished on a polypropylene brush until shiny before use. The solution of LPSCl and polymer was prepared as follows. LPSCl was dissolved in ethanol at a concentration of 0.2 g/mL. PEO was dissolved in acetonitrile and PPO was dissolved in toluene at a concentration of 1, 5, and 10 mg/mL for both polymers. Then, the LPSCl solution and polymer solution were co-precipitated in centrifuge tubes. The mixture was centrifuged at 6000 rpm for 10 min. The overall processing time of LPSCl, including dissolution, precipitation, and separation steps, was controlled to be within 15 min to minimize the negative effect of EtOH on LPSCl. The solvent was then discarded, and the precipitate was dried under a vacuum at room temperature overnight. For the PPO–LPSCl composite, the sample was further dried at 60 °C for at least 2 h. Finally, the composites were grounded to yield a gray powder composite. Elemental analysis results: PEO–LPSCl composites: C 1.00%, H 0.22%, S 57.55% (1 mg/mL PEO solution), C 2.32%, H 0.71%, S 51.21% (5 mg/mL PEO solution), C 5.88%, H 1.04%, S 50.55% (10 mg/mL PEO solution). PPO–LPSCl composites: C 2.58%, H 0.33%, S 55.81% (1 mg/mL PPO solution), C 6.75%, H 1.77%, S 47.90% (5 mg/mL PPO solution), C 9.44%, H 2.01%, S 46.31% (10 mg/mL PPO solution). According to the elemental analysis results, the polymer content in the composites is confirmed as 2, 8, and 12 wt % from the solution–precipitation method using polymer solutions (i.e., PEO in acetonitrile and PPO in toluene) of 1, 5, and 10 mg/mL, respectively.

### 2.2 Polymer Composites via Solvent–Dispersion and Mechanical Mixing

Ten milligrams per milliliters of PEO was prepared in acetonitrile, and 10 mg/mL of PPO was prepared in toluene. Three composites of 2, 8, and 12 wt % were prepared by simply mixing LPSCl and polymer solution at an exact polymer weight percent content. The mixture was then dried under a vacuum at room temperature overnight. Finally, the dispersion composites were grounded to yield a gray powder composite. The LPSCl powder and the polymer powder were mixed using a mortar and pestle for a mechanically mixed composite.

### 2.3 Pellet Preparation

All pellets were pressed in a stainless steel pellet pressing die-set 6 mm in diameter. Briefly, every pellet was pressed using 50 mg of powder composite and 350 MPa was applied and held for at least 30 s for each pellet, providing a thickness of 1.2 mm. For the PEO composite, the pellets were annealed at 180 °C in a vacuum oven, while PPO composite pellets were annealed at 400 °C under an argon atmosphere overnight.

### 2.4 Material Characterization

A Thermo Fisher FlashSmart elemental analyzer was used to analyze the composites’ polymer contents (C, H, N, and S). X-ray diffraction (XRD) of the composites was performed using Bruker-XRD D2 Phaser equipped with an airtight holder and a knife-edge beam stop. The microstructure and morphology of materials were investigated using a scanning electron microscope (SEM, JEOL JSM-7500F). Elemental distributions of the composites were confirmed via a transmission electron microscope (TEM)-based energy-dispersive X-ray spectroscopy (EDS) analysis. The high-angle annular dark-field scanning TEM (HAADF-STEM) and EDS elemental maps were collected on FEI ThemIS 60–300 TEM. A Bruker SuperX EDS detector, equipped on the TEM, prevents potential sample damages during elemental mapping by reducing the signal acquiring time compared with the conventional TEM. For the sample preparation, the composites were dispersed into hexane solvent by sonication and then drop cast on Cu grids (lacey carbon 400-mesh Cu grid, Ted Pella, Inc.). Subsequently, the samples were transferred to the TEM for microstructural observation using an airtight sample transfer holder (Model 648, Gatan Inc.).

### 2.5 Mechanical Test

A nanoindentation test was employed to evaluate the mechanical properties of the LPSCl–polymer composites. A Hysitron TI 950 TriboIndenter system and a Berkovich tip were used for the test. The machine compliance was calibrated with a polycarbonate standard sample. All composite pellets were sealed in a plastic bag under an inert gas (Ar) atmosphere; subsequently, the packages were transferred into light mineral oil for the test. After that, the plastic bag was removed, and the pellet was placed on a stainless steel stub with a super glue gel and then magnetically attached to the instrument. We confirmed that all processes were operated with the pellet immersed in the oil to avoid oxygen and moisture exposure. The indenter was held for 4 s at the maximum load for the measurement. The load rate is 600 nm/s with a load control of 9 mN. The reduced Young’s modulus and hardness values were determined based on the Oliver–Pharr method. During the test, the indenter was contiguously immersed in the oil and kept the probe from approaching the pellet surface. Therefore, mineral oil’s surface tension or buoyant force will not influence the results. The force–distance curves and residual hardness impressions of all samples are displayed in the Supporting Information (Figures S1 and S2).

### 2.6 Electrochemical Test

Metrohm Autolab (FRA32M-impedance analysis) was used to measure the ionic conductivity using a Swagelok-type cell, which was built as follows: indium (100 μm)//sample pellet (1.2 mm)//indium (100 μm). For the lithium striping–plating performance test, the Swagelok-type cell was built as Li (100 μm)//sample pellet (1.2 mm)//Li (100 μm). Galvanostatic cycling was performed at a rate of 0.1 mAh/cm^2^, in which a 0.48 μm thick piece of lithium will be striped/plated back and forth. The cell voltage was monitored over time. The rapid decrease in voltage was regarded as a sign of the dendrite formation across the pellet. The pressure applied for electrochemical testing was provided by the internal springs of the Swagelok-type cell, which was estimated to be 0.2 MPa. All electrochemical tests were performed at 25 ± 2 °C

## 3 Results and Discussion

**Figure 2:**
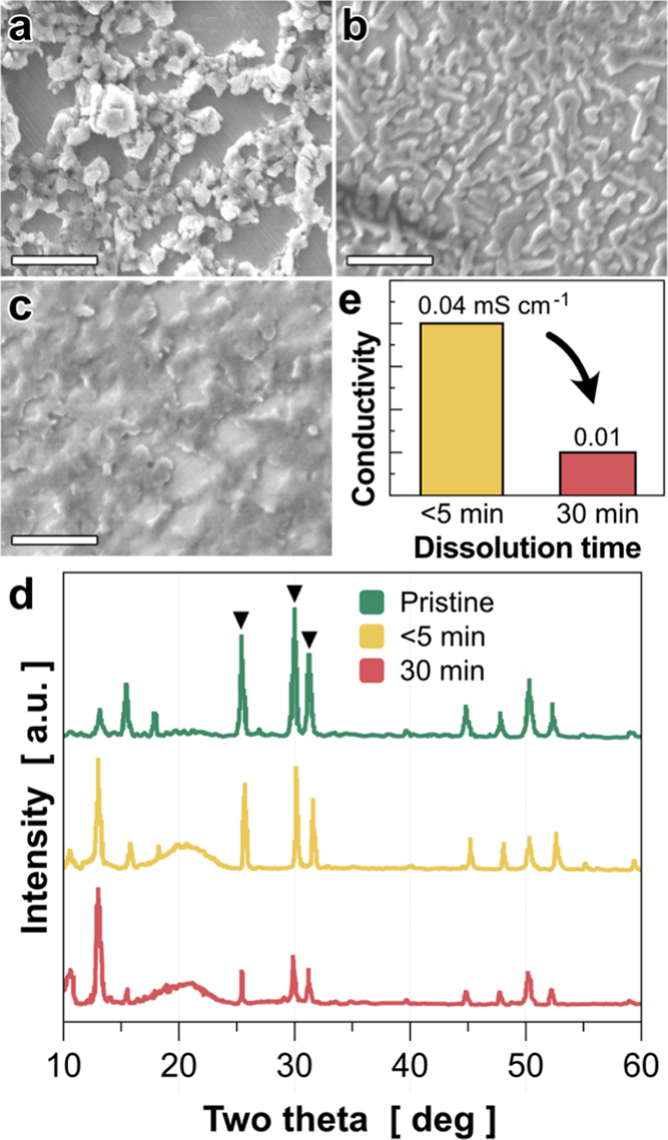
Morphological and structural changes with dissolution time in EtOH. (a–c) SEM images of (a) pristine LPSCl powder and (b) LPSCl precipitate after dissolution in EtOH for 5 min and (c) after dissolution for 30 min. Scale bars in SEM images are 10 μm. (d) XRD results of LPSCl with a different dissolution time in EtOH. As time increases, the intensities of the representative LPSCl peaks (denoted as a triangle) decreases. Please note that the sharp peak at ∼13° and the bump peak at ∼20° are from an airtight XRD holder used in yellow and red patterns. (e) Ionic conductivities of LPSCl with different dissolution times. Note that a shorter dissolution time of LPSCl is preferred to exhibit a higher conductivity.

A novel solution–precipitation approach was employed to prepare LPSCl–polymer composites using polyethylene oxide (PEO) and poly(phenylene oxide) (PPO) polymers. PEO can provide conductive ion channels via ethylene oxide and Li-ion complex.^23^ In contrast, PPO is one of the thermally stable polymers due to its sp^2^ character, which can offer a post-thermal treatment at high temperatures.^24^ According to the literature, ethanol (EtOH) can be used as a solvent to infiltrate LPSCl into a porous cathode composite. Still, the overall liquid process must be done within 1 h due to the time-sensitive property of the LPSCl in the solvent.^25^ Thus, we first investigated the effect of dissolution time on the LPSCl properties, as shown in Figure 2. SEM images (Figure 2a–c) show the decrease in the grain size of the SE with the dissolution time in EtOH, and the well-defined particle shape was lost at a longer dissolution time (30 min). In addition, XRD results show a decrease in the signal for both cases compared to the pristine LPSCl, but the 30 min sample shows the lowest intensity of XRD peaks among the samples (Figure 2d). Please note that the sharp peak at ∼13° and the bump peak at ∼20° come from an airtight XRD holder used in yellow and red patterns in Figure 2d. A decrease in the ionic conductivity with the dissolution time is also observed (Figure 2e). The 30 min sample shows a comparatively lower conductivity (0.01 mS/cm) than the 5 min sample (0.04 mS/cm). These results suggest that the dissolution of LPSCl in EtOH will affect the overall crystal structure and ionic conductivity of the recovered precipitates and those properties are strongly dependent on the dissolution time. Thus, the solution–precipitation method requires the minimum exposure of LPSCl to EtOH to minimize the depletion of the crystal structure of LPSCl and may require additional heat treatment to recover some crystal structure and ionic conductivity of LPSCl.

To evaluate the proposed solution–precipitation method, we comparatively investigate the morphological and mechanical properties of the LPSCl–polymer composites with the other preparation methods. First, we investigate the morphology of LPSCl–PEO composites using SEM images (see Figure S3 in the Supporting information). Regardless of the polymer content, all composites displayed similar morphology with crystal grain sizes between 1 and 10 μm. Unlike the size of LPSCl, which decreased after the precipitation without polymer (Figure 2), the feature size of the polymer composite appears to be preserved when the precipitation was performed in the polymer solution (Figure S3). This result suggests that the polymer promotes LPSCl precipitation and possibly limits ethanol accessibility to LPSCl.

**Figure 3:**
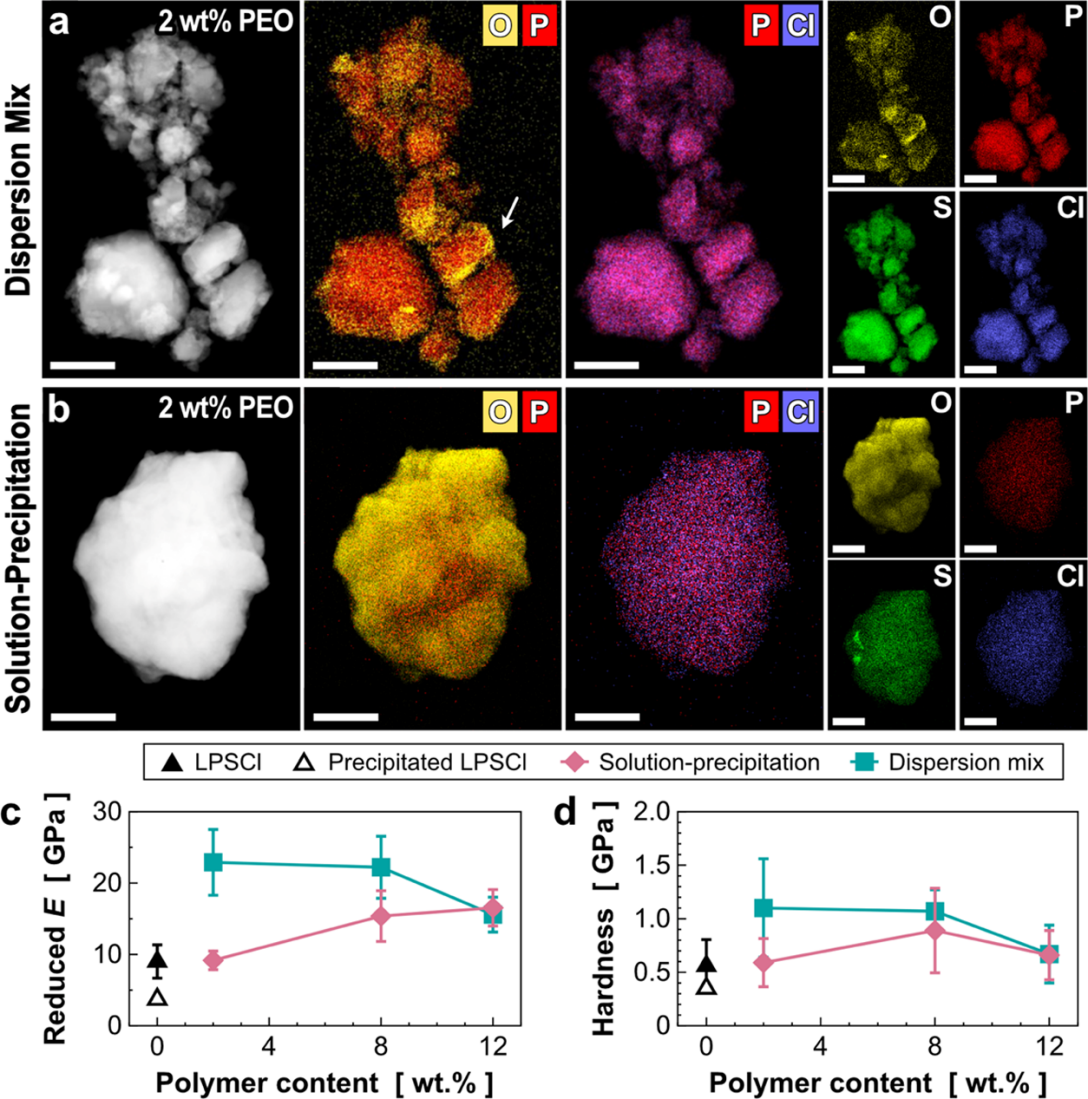
Chemical and mechanical evaluations of the LPSCl–PEO composite. (a, b) Elemental mappings of (O, P, S Cl) in the LPSCl–PEO composites with 2 wt % polymer content. Note that the composites are prepared by two different methods: (a) dispersion mix method and (b) solution–precipitation method. (c, d) Comparison of (c) reduced Young’s moduli and (b) hardnesses obtained from nanoindentation tests. An effect on the polymer contents is also investigated. Mechanical properties of 2, 8, and 12 wt % PEO and LPSCl composites are compared as well as those of the pristine LPSCl and the precipitated LPSCl. Scale bars in TEM images are 1 μm.

Because we could not identify the polymer phase in SEM images, we performed TEM-EDS analyses to track how the polymer distributes on the composites. For the TEM analyses, we used an airtight sample transfer TEM holder to avoid any potential degradation of the composite due to air exposure (For details, see Section 2). Figure 3 shows the comparative results between the LPSCl–PEO composites by the dispersion method (Figure 3a) and the composites via the solution–precipitation method (Figure 3b). Considering that all sample preparation procedures were conducted without air exposure, oxygen (O) elemental distribution in the EDS maps is expected to represent the presence of PEO along with the LPSCl phase. To compare the elemental distribution clearly, we put bi-elemental maps of P–O and P–Cl right next to each STEM-HAADF image. For the 2 wt % polymer composites prepared by the dispersion mix method, a nonuniform distribution of PEO is clearly observed as shown in Figure 3a (in bi-elemental maps of P–O). For example, we can clearly observe the phase separation of PEO-rich and LPSCl-rich domains in Figure 3a. In contrast, the solution–precipitation method creates a uniform distribution of PEO in LPSCl composites, where no obvious phase separation between PEO-rich and LPSCl-rich domains is identified (see bi-elemental maps of P–O in Figure 3b). These results confirm that the solution–precipitation method is better than the dispersion mix one for a uniform distribution of the polymer phase at a submicron-scale level.

The mechanical properties of the LPSCl–PEO composites were evaluated via nanoindentation tests (Figure 3c,d). As expected, the reduced Young’s modulus of the dispersion composites decreases as the polymer content increases (Figure 3c). This result is likely attributable to the phase separation between PEO and LPSCl, causing poor interactions between the two phases. In contrast, the reduced Young’s modulus of the composites prepared via the solution–precipitation method increases as the polymer content increases, confirming the importance of polymer distribution in SE composite for better mechanical reinforcement. For the hardness, the trend of both types of samples is similar, and the values decrease when the polymer content increases (Figure 3d). This is an understandable result because PEO is a soft material: increasing PEO content will correspondingly lower the hardness of the composite materials. The hardness of the 8 wt % solution–precipitation composites seems to increase, but the value is insignificant given the relatively large error scale.

Interestingly, composites by the solution–precipitation method exhibit lower mechanical properties than those prepared via dispersion mix. This difference is more clearly observed with a smaller polymer content. It can be explained that the difference can be originated from the structural degradation of LPSCl during the solution–precipitation process, as previously shown in Figure 2. The dispersion method was performed using the pristine LPSCl, which has improved crystallinity and mechanical properties. In contrast, the degradation of the solution–precipitation method was inevitable even in the shortest process time (<15 min). The most general way to recover the crystallinity of LPSCl is annealing at a high temperature, typically above 400 °C.^26^ To find a proper temperature to anneal the composite without decomposing the polymers, we preliminarily tested the thermal decomposition using TGA for PEO (see Figure S4 in the Supporting Information). The decomposition temperature onset for PEO was around 350 °C. Because there was no significant improvement in the crystallinity as well as the conductivity of the LPSCl–PEO composite if it was annealed at lower than 350 °C, we annealed the LPSCl–PEO composite at 180 °C. It might be difficult to achieve a high crystallinity of LPSCl in typical ways in the composite with PEO.

**Figure 4:**
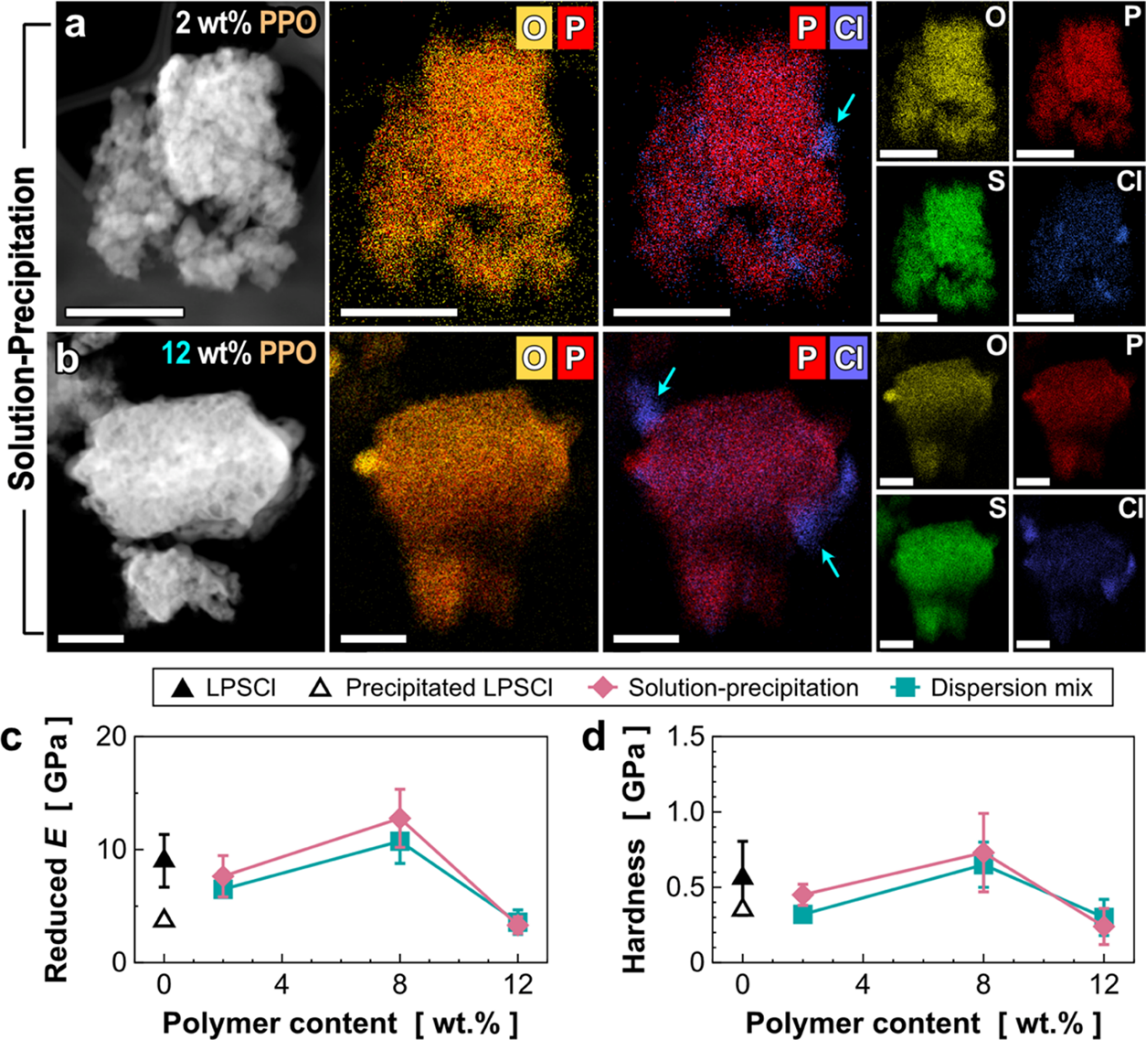
Chemical and mechanical evaluations of the LPSCl–PPO composite. (a,b) Elemental mappings of (O, P, S Cl) in the LPSCl–PPO composites with different polymer contents: (a) 2 wt % and (b) 12 wt %. Note that the observed LiCl phase regions were denoted by blue arrows. (c, d) Comparison of (c) reduced Young’s modulus and (d) hardness of the LPSCl–PPO composites. The composite samples were annealed at 400°C before the mechanical tests. Scale bars in TEM images are 500 nm.

Accordingly, we investigated the properties of the LPSCl–PPO composites prepared by the solution–precipitation method. Owing to the higher thermal stability of polyphenylene oxide (PPO) than that of the PEO, we expected to achieve a higher crystallinity of LPSCl by allowing a higher temperature (400 °C) for the annealing process. The observed morphology of the LPSCl–PPO composite via SEM analysis is quite similar to that of the LPSCl–PEO composites; their crystal grain size ranges from 1 to 10 μm, and the polymer phase is not distinguishable in SEM images (see Figure S5 in the Supporting Information). Thus, we performed elemental mapping using TEM-EDS to monitor the polymer distribution in the composites. Figure 4a,b shows a good distribution of the O element, which represents the polymer phase, and the P element, which belongs to the LPSCl phase, regardless of the PPO content. There are also some small portions of the free polymer phase observed in the image, which is sometimes seen in high polymer-loaded composites (both PEO and PPO). As a downside of this method, we found the segregated Cl region, expected to be LiCl, indicating LPSCl decomposition during the process (denoted by blue arrows in Figure 4a,b). The decomposition could have originated from the dissolution of LPSCl in ethanol where some of the crystals are completely dissolved and cannot reform back to the same structure. This effect can be minimized by the reduction of ethanol exposure time as mentioned earlier. For the mechanical properties of the LPSCl–PPO composites, both the reduced Young’s modulus and the hardness of the PPO composites are shown in Figure 4c,d. In the case of the composites from the solution–precipitation method, the composites with 2 and 8 wt % of PPO show improved mechanical strength compared to the precipitated LPSCl, but no significant improvement is observed when compared to the pristine LPSCl. In addition, the mechanical reinforcement by PPO is negligible compared to the PEO system (Figure 3). Similarly, the dispersion method did not show any noticeable improvement in mechanical strength when compared to the pristine LPSCl, which is opposite to the LPSCl–PEO composites. This result could be due to the incompatibility between the polar inorganic salt (LPSCl) and a much less polar organic polymer (PPO), which might lower the interactions between SE and the polymer, causing the degradation of mechanical properties.

The effects of polymer species and their content on the Li conductivity were evaluated using electrochemical impedance spectroscopy (EIS). The result summary is shown in Figure S6 in the Supporting Information. The conductivity decreases when the PEO content increases for both dispersion and solution–precipitation methods. Note that the solution–precipitation method shows a more significant decrease in conductivity as the polymer content increases (10^–2^ vs 1 mS/cm of pristine LPSCl). The low conductivity could be attributed to the LPSCl forming smaller crystals or an amorphous phase after the solution–precipitation reaction. Therefore, thermal treatment at a higher temperature is required to improve the crystallinity of LPSCl solid electrolytes. For the LPSCl–PPO composites, the mechanical mix and the dispersion samples displayed similar conductivity and trends in which the increase in the polymer content lowers the conductivity of the materials. Interestingly, the conductivity of PPO composites from the solution–precipitation method exhibits a more negligible effect on the polymer content (from 2 to 12 wt %). This could be due to the well-distributed polymer in which there is no large polymer domain to block ion-conductive channels. However, significantly lower conductivity of the solution–precipitated composite compared to the other two methods was observed in the case of 2 and 8 wt % PPO content. It is likely that the uniform distribution of the polymer prevents the crystallization of LPSCl and/or the exposure of LPSCl to ethanol decomposes LPSCl to other irreversible nonconductive salts like LiCl. Typically, LPSCl is known to undergo hydrolysis with water, causing irreversible loss of sulfur by forming hydrogen sulfide (H_2_S). In ethanol, we expect that the LPSCl may undergo ethanolysis, releasing H_2_S but at a much slower rate than water since the available proton of ethanol to form H_2_S is less active than that of water (p*K*_a_ of 16 of ethanol vs p*K*_a_ of 14 of water).

**Figure 5:**
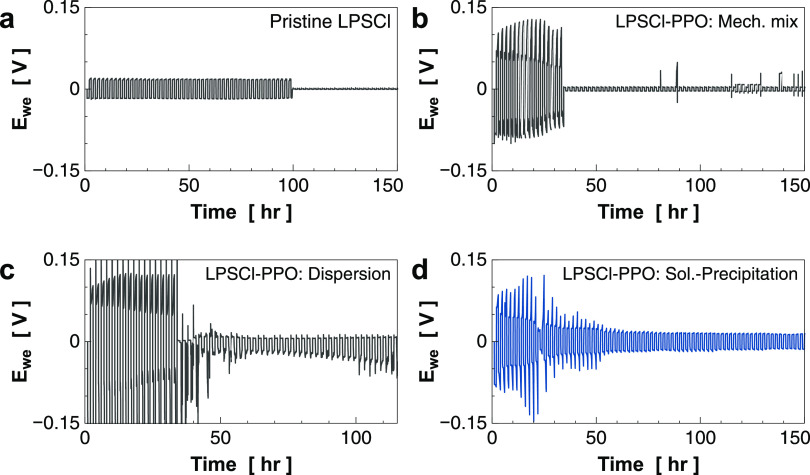
Lithium striping/plating cycle properties of (a) pristine LPSCl and LPSCl–PPO composite prepared by (b) mechanical mix, (c) dispersion mix, and (d) solution–precipitation methods.

Finally, we investigated the stability of dendrite formation on the LPSCl–PPO composites via repetitive Li striping/plating tests over time at a constant current with a rate of 0.1 mAh/cm^2^. We tested four materials: three types of LPSCl–PPO composite from solution–precipitation, dispersion mix, mechanical mix, and the pristine LPSCl. The 12 wt % PPO–LPSCl composite was selected from each method for the test because they exhibit comparable ionic conductivity to each other to avoid the effect of ionic conductivity on the Li striping/plating cycles. The cycle results are shown in Figure 5. The pristine LPSCl shows smooth Li striping–plating with a constant voltage for at most 50 cycles, followed by the voltage drop, indicating the penetration of dendrite across SE. In contrast, the cycle of all three composites displays a high overpotential at the initial few cycles and tends to decrease after each cycle. These events could be contributed to the interface issues between Li metal and low ion-conductive PPO in the composite, which can increase interface resistance. During the cycle, the interface becomes reconstructed, resulting in a decrease in interface resistance. This explains the lowering of the overpotential during the cycle process. For both cells from mechanical mixing and dispersion composites, less than 20 cycles are observed before the voltage drop, implying lower stability toward dendrite formation than the pristine LPSCl. Impressively, the cell made from the LPSCl–PPO composite via the solution–precipitation displays incredibly high stability over 100 cycles without an observed voltage drop. Although the mechanical strength of the composite is lower than the pristine one, the stability toward dendrite formation can be improved, which is likely attributable to the lowering of electronic conductivity by uniformly distributing the electronic insulator, PPO, in SE. These results further confirmed the importance of the homogeneous polymer phase distribution in the composite to sufficiently suppress the electronic conductivity.

## 5 Conclusions

LPSCl–polymer composites via the solution–precipitation process were successfully prepared. The uniform polymer distribution in the composite was confirmed by TEM-EDS mapping: no separated polymer domains were observed, while clear large polymer domains were found in the case of the dispersion method. The nanoindentation was used to evaluate the mechanical strength of all composites. The polymer and SE compatibility is another factor to be concerned about because it contributes to the overall mechanical reinforcement in the composites. LPSCl–PEO via the solution–precipitation method exhibits a linear relationship between reduced Young’s modulus and wt % polymer content, while the dispersion composites display an inverse trend. On the other hand, there was no noticeable mechanical reinforcement observed in the LPSCl–PPO composites. In terms of ionic transport properties, the conductivity of up to 0.3 mS/cm of the 12 wt % PPO–LPSCl solution–precipitation composite is achieved. The SE composite survives over 100 cycles of Li striping–plating test longer than pristine LPSCl and other composites from mechanical mixing and dispersion methods. These results support the potential of the solution–precipitation method for preparing two phases’ composites with highly homogeneous mixing of the two phases. The PPO–LPSCl composites are investigated as a potential SE for all-solid-state lithium battery applications.

## Supporting Information Available

Force–distance curves from nanoindentation; residual hardness impressions; SEM images of LPSCl–PEO and LPSCL–PPO composites; TGA results; conductivity of PEO-–LPSCl and PPO–LPSCl composites; and accumulated lithium capacity during striping/plating test (PDF)

## References

[ref1] Diaz L. B.; He X.; Hu Z.; Restuccia F.; Marinescu M.; Barreras J. V.; Patel Y.; Offer G.; Rein G. Review—Meta-Review of Fire Safety of Lithium-Ion Batteries: Industry Challenges and Research Contributions. J. Electrochem. Soc. 2020, 167, 090559 10.1149/1945-7111/aba8b9.

[ref2] Schnell J.; Knörzer H.; Imbsweiler A. J.; Reinhart G. Solid versus Liquid—A Bottom-Up Calculation Model to Analyze the Manufacturing Cost of Future High-Energy Batteries. Energy Technol. 2020, 8, 1901237 10.1002/ente.201901237.

[ref3] Monroe C.; Newman J. The Impact of Elastic Deformation on Deposition Kinetics at Lithium/Polymer Interfaces. J. Electrochem. Soc. 2005, 152, A396 10.1149/1.1850854.

[ref4] Brissot C.; Rosso M.; Chazalviel J.-N.; Lascaud S. Dendritic Growth Mechanisms in Lithium/Polymer Cells. J. Power Sources 1999, 81–82, 925–929. 10.1016/S0378-7753(98)00242-0.

[ref5] Byeon Y.-W.; Kim H. Review on Interface and Interphase Issues in Sulfide Solid-State Electrolytes for All-Solid-State Li-Metal Batteries. Electrochem 2021, 2, 452–471. 10.3390/electrochem2030030.

[ref6] LaCoste J. D.; Zakutayev A.; Fei L. A Review on Lithium Phosphorus Oxynitride. J. Phys. Chem. C 2021, 125, 3651–3667. 10.1021/acs.jpcc.0c10001.

[ref7] Kim S.; Oguchi H.; Toyama N.; Sato T.; Takagi S.; Otomo T.; Arunkumar D.; Kuwata N.; Kawamura J.; Orimo S. A Complex Hydride Lithium Superionic Conductor for High-Energy-Density All-Solid-State Lithium Metal Batteries. Nat. Commun. 2019, 10, 1081 10.1038/s41467-019-09061-9. 30842419 PMC6403359

[ref8] Mishra M.; Hsu C.-W.; Rath P. C.; Patra J.; Lai H.-Z.; Chang T.-L.; Wang C.-Y.; Wu T.-Y.; Lee T.-C.; Chang J.-K. Ga-Doped Lithium Lanthanum Zirconium Oxide Electrolyte for Solid-State Li Batteries. Electrochim. Acta 2020, 353, 136536 10.1016/j.electacta.2020.136536.

[ref9] Lian P.-J.; Zhao B.-S.; Zhang L.-Q.; Xu N.; Wu M.-T.; Gao X.-P. Inorganic Sulfide Solid Electrolytes for All-Solid-State Lithium Secondary Batteries. J. Mater. Chem. A 2019, 7, 20540–20557. 10.1039/C9TA04555D.

[ref10] Han F.; Westover A. S.; Yue J.; Fan X.; Wang F.; Chi M.; Leonard D. N.; Dudney N. J.; Wang H.; Wang C. High Electronic Conductivity as the Origin of Lithium Dendrite Formation within Solid Electrolytes. Nat. Energy 2019, 4, 187–196. 10.1038/s41560-018-0312-z.

[ref11] Cheng E. J.; Sharafi A.; Sakamoto J. Intergranular Li Metal Propagation through Polycrystalline Li6.25Al0.25La3Zr2O12 Ceramic Electrolyte. Electrochim. Acta 2017, 223, 85–91. 10.1016/j.electacta.2016.12.018.

[ref12] Yonemoto F.; Nishimura A.; Motoyama M.; Tsuchimine N.; Kobayashi S.; Iriyama Y. Temperature Effects on Cycling Stability of Li Plating/Stripping on Ta-Doped Li7La3Zr2O12. J. Power Sources 2017, 343, 207–215. 10.1016/j.jpowsour.2017.01.009.

[ref13] Tsai C.-L.; Roddatis V.; Chandran C. V.; Ma Q.; Uhlenbruck S.; Bram M.; Heitjans P.; Guillon O. Li7La3Zr2O12 Interface Modification for Li Dendrite Prevention. ACS Appl. Mater. Interfaces 2016, 8, 10617–10626. 10.1021/acsami.6b00831. 27029789

[ref14] Porz L.; Swamy T.; Sheldon B. W.; Rettenwander D.; Frömling T.; Thaman H. L.; Berendts S.; Uecker R.; Carter W. C.; Chiang Y.-M. Mechanism of Lithium Metal Penetration through Inorganic Solid Electrolytes. Adv. Energy Mater. 2017, 7, 1701003 10.1002/aenm.201701003.

[ref15] Chen H.; Adekoya D.; Hencz L.; Ma J.; Chen S.; Yan C.; Zhao H.; Cui G.; Zhang S. Stable Seamless Interfaces and Rapid Ionic Conductivity of Ca–CeO2/LiTFSI/PEO Composite Electrolyte for High-Rate and High-Voltage All-Solid-State Battery. Adv. Energy Mater. 2020, 10, 2000049 10.1002/aenm.202000049.

[ref16] Chen H.; Zheng M.; Qian S.; Ling H. Y.; Wu Z.; Liu X.; Yan C.; Zhang S. Functional Additives for Solid Polymer Electrolytes in Flexible and High-Energy-Density Solid-State Lithium-Ion Batteries. Carbon Energy 2021, 3, 929–956. 10.1002/cey2.146.

[ref17] Yu X.; Manthiram A. A Review of Composite Polymer-Ceramic Electrolytes for Lithium Batteries. Energy Storage Mater. 2021, 34, 282–300. 10.1016/j.ensm.2020.10.006.

[ref18] Yao P.; Yu H.; Ding Z.; Liu Y.; Lu J.; Lavorgna M.; Wu J.; Liu X. Review on Polymer-Based Composite Electrolytes for Lithium Batteries. Front. Chem. 2019, 7, 522 10.3389/fchem.2019.00522. 31440498 PMC6694289

[ref19] Whiteley J. M.; Taynton P.; Zhang W.; Lee S.-H. Ultra-Thin Solid-State Li-Ion Electrolyte Membrane Facilitated by a Self-Healing Polymer Matrix. Adv. Mater. 2015, 27, 6922–6927. 10.1002/adma.201502636. 26421754

[ref20] Zhang J.; Zheng C.; Lou J.; Xia Y.; Liang C.; Huang H.; Gan Y.; Tao X.; Zhang W. Poly(Ethylene Oxide) Reinforced Li6PS5Cl Composite Solid Electrolyte for All-Solid-State Lithium Battery: Enhanced Electrochemical Performance, Mechanical Property and Interfacial Stability. J. Power Sources 2019, 412, 78–85. 10.1016/j.jpowsour.2018.11.036.

[ref21] Wang S.; Zhang X.; Liu S.; Xin C.; Xue C.; Richter F.; Li L.; Fan L.; Lin Y.; Shen Y.; Janek J.; Nan C.-W. High-Conductivity Free-Standing Li6PS5Cl/Poly(Vinylidene Difluoride) Composite Solid Electrolyte Membranes for Lithium-Ion Batteries. J. Mater. 2020, 6, 70–76. 10.1016/j.jmat.2019.12.010.

[ref22] Fu C.; Venturi V.; Kim J.; Ahmad Z.; Ells A. W.; Viswanathan V.; Helms B. A. Universal Chemomechanical Design Rules for Solid-Ion Conductors to Prevent Dendrite Formation in Lithium Metal Batteries. Nat. Mater. 2020, 19, 758–766. 10.1038/s41563-020-0655-2. 32341510

[ref23] Xue Z.; He D.; Xie X. Poly(Ethylene Oxide)-Based Electrolytes for Lithium-Ion Batteries. J. Mater. Chem. A 2015, 3, 19218–19253. 10.1039/C5TA03471J.

[ref24] McKeen L. W. High-Temperature/High-Performance Polymers. In The Effect of Long Term Thermal Exposure on Plastics and Elastomers; William Andrew Publishing: Oxford, 2014; pp 209–238.

[ref25] Kim D. H.; Oh D. Y.; Park K. H.; Choi Y. E.; Nam Y. J.; Lee H. A.; Lee S.-M.; Jung Y. S. Infiltration of Solution-Processable Solid Electrolytes into Conventional Li-Ion-Battery Electrodes for All-Solid-State Li-Ion Batteries. Nano Lett. 2017, 17, 3013–3020. 10.1021/acs.nanolett.7b00330. 28362097

[ref26] Kim D. H.; Lee Y.-H.; Song Y. B.; Kwak H.; Lee S.-Y.; Jung Y. S. Thin and Flexible Solid Electrolyte Membranes with Ultrahigh Thermal Stability Derived from Solution-Processable Li Argyrodites for All-Solid-State Li-Ion Batteries. ACS Energy Lett. 2020, 5, 718–727. 10.1021/acsenergylett.0c00251.

