# Supplementary material for: Lithium Phosphorus Sulfide Chloride–Polymer Composite via the Solution–Precipitation Process for Improving Stability toward Dendrite Formation of Li-Ion Solid Electrolyte
Source: ACS Appl Mater Interfaces. 2023 Feb 24;15(9):11723–30. doi: 10.1021/acsami.2c21302 (PMC9999344; doi:10.1021/acsami.2c21302)
Supplement: Supplementary file 1 — am2c21302_si_001.pdf [file am2c21302_si_001.pdf]

Supporting information

Lithium Phosphorus Sulfide Chloride-Polymer Composite via Solution-Precipitation Process for Improving Stability toward Dendrite Formation of Li-Ion Solid Electrolyte

Piyachai Khomein,[a,b,^] *Young-Woon Byeon,*[c,^] *Dongye Liu,*[d] *Jin Yu,*[b,e] *Andrew M. Minor,*[c,d,f] *Haegyeom Kim,*[c,*] *Gao Liu*[b,*]

[a] Division of Nuclear Medicine, Department of Radiology, Faculty of Medicine, Chulalongkorn University, Bangkok, 10330, Thailand

[b] Energy Storage and Distributed Resources Division, Energy Technologies Area, Lawrence Berkeley National Laboratory, Berkeley, California 94720, United States

[c] Materials Sciences Division, Lawrence Berkeley National Laboratory, Berkeley, California 94720, United States

[d] Department of Materials Science and Engineering, University of California, Berkeley, California, 94720, United States

[e] Department of Chemical & Biomolecular Engineering, University of California, Berkeley, California, 94720, United States

[f] National Center for Electron Microscopy, The Molecular Foundry, Lawrence Berkeley National Laboratory, Berkeley, California 94720, United States

^ Equal Authorship

*Corresponding authors: haegyumkim@lbl.gov (H.K.); gliu@lbl.gov (G. L.)

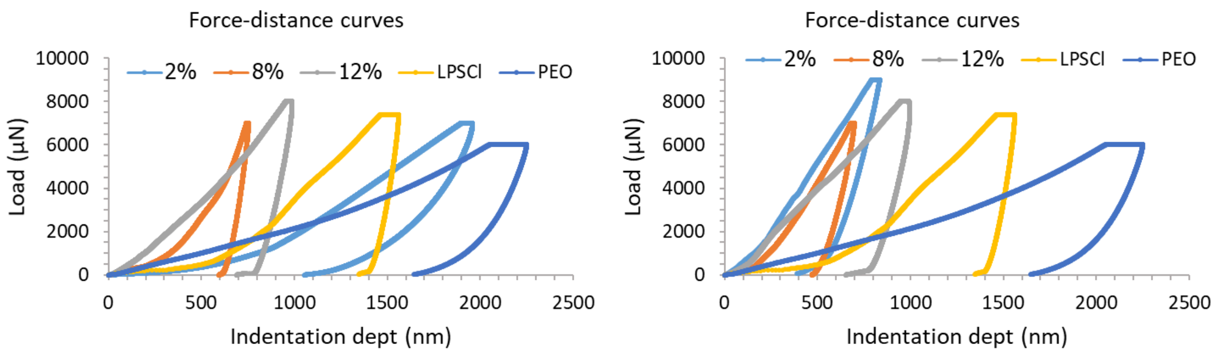

Figure S1. Force-distance curves from nanoindentation of LPSCl-PPO composites from dispersion method (left) and solution-precipitation method (right) compared to pristine LPSCl and PEO

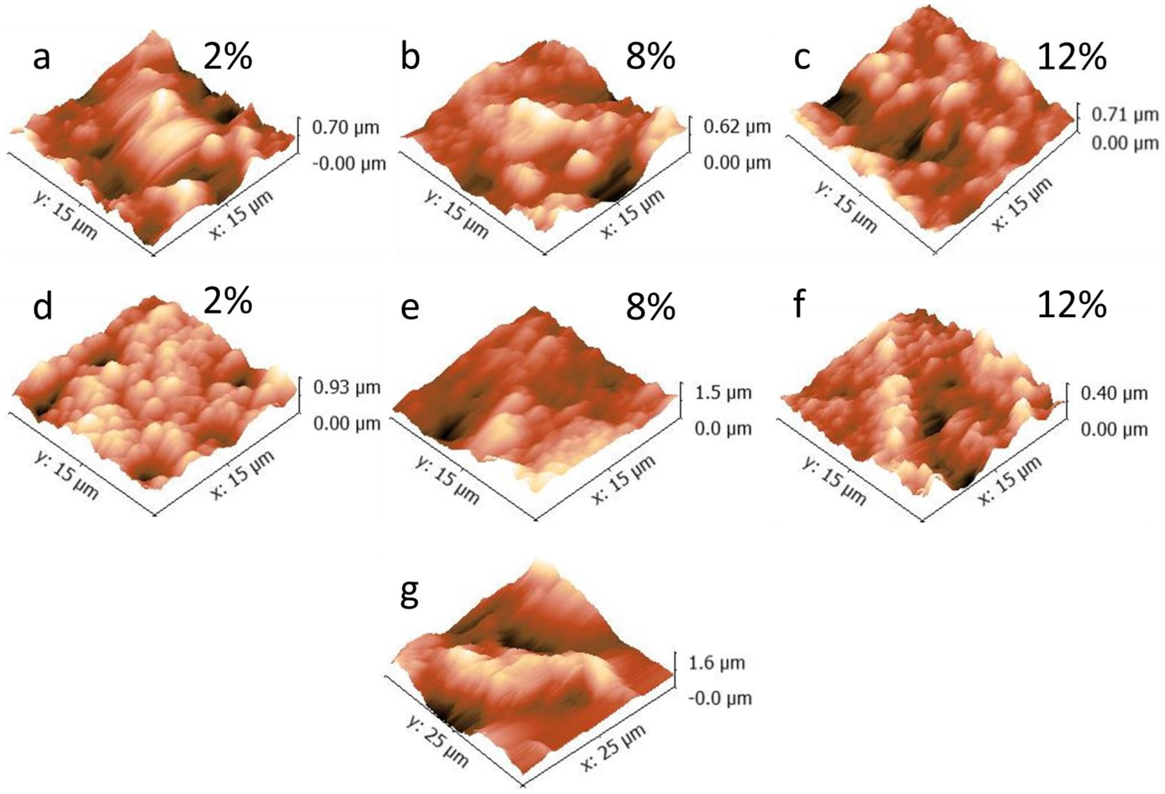

Figure S2. Residual hardness impressions of LPSCl-PPO composites form dispersion method (a-c), solution-precipitation method (d-e), and pristine LPSCl (g)

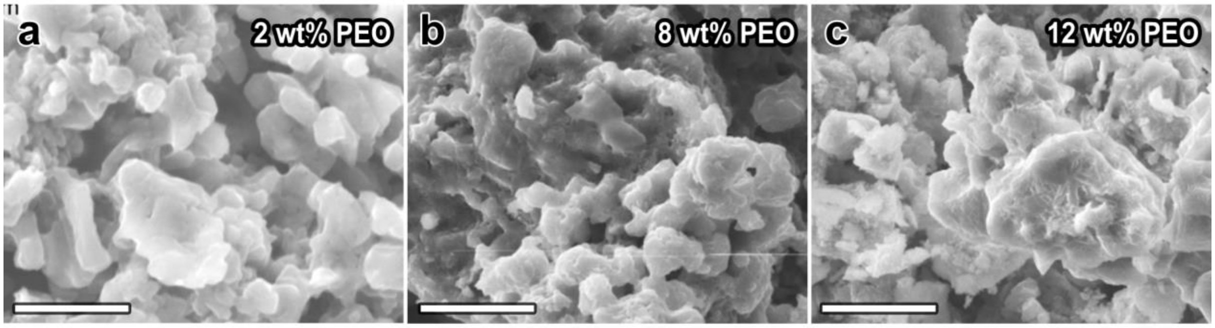

Figure S3. Scanning electron microscope (SEM) images of LPSCl-PEO composites with the polymer content of 2%w (a), 8%wt (b), and 12%wt (c) (from left to right).

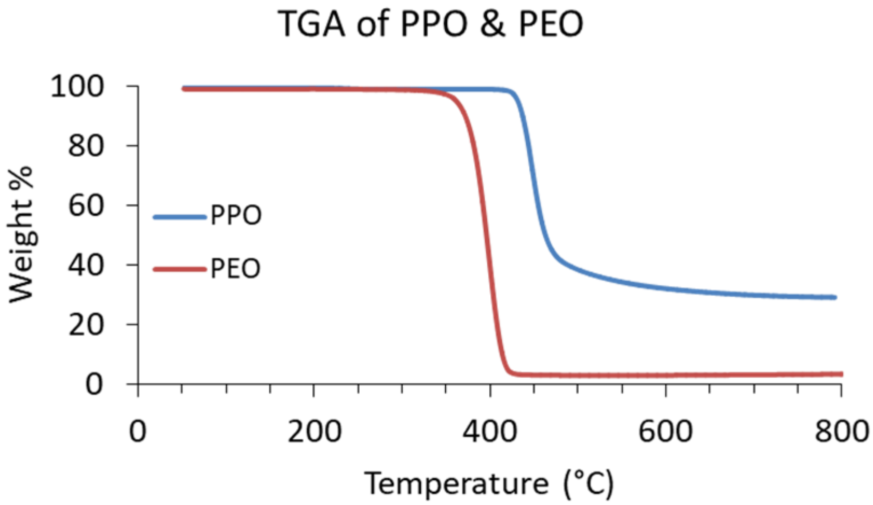

Figure S4. TGA results of PEO and PPO polymers. The tests were conducted in N_2 gas environment.

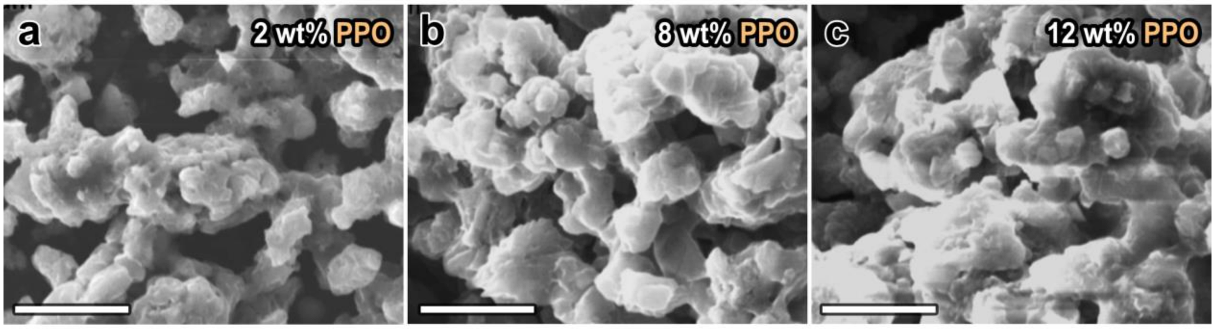

Figure S5. Scanning electron microscope (SEM) images of LPSCl-PPO composites with the polymer content of 2%w (a), 8%wt (b), and 12% wt (c) (from left to right).

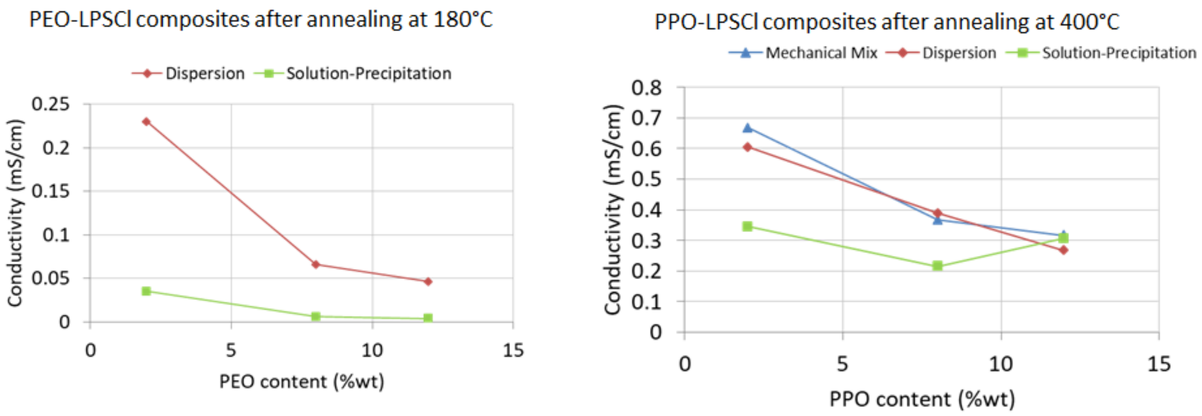

Figure S6. Conductivity of PEO-LPSCl composites after 180°C annealing (left) and PPO-LPSCl composites after 400°C (right)

Table S1. Summary of lithium plating/striping of pristine LPSCl and LPSCl-PPO composite prepared by mechanical mix, dispersion mix, and solution-precipitation methods.

Sample	Pristine LPSCl	LPSCl-PPO composites		
		Mechanical mix	Dispersion mix	Our method
Total hour before voltage drop (h)	98	34	36	>150
Total lithium striping/plating (mg)	12.7	4.4	4.7	>19.5
Accumulated lithium capacity (mAh/cm^2)	9.8	3.4	3.6	>15
